# The Traumatic Brain Injury Model Systems National Database: A Review of Published Research

**DOI:** 10.1089/neur.2020.0047

**Published:** 2021-03-12

**Authors:** Samantha Tso, Ashirbani Saha, Michael D. Cusimano

**Affiliations:** ^1^ Division of Neurosurgery, St. Michael's Hospital, Toronto, Ontario, Canada.; ^2^ Li Ka Shing Knowledge Institute, St. Michael's Hospital, Toronto, Ontario, Canada.; ^3^ Department of Surgery, University of Toronto, Toronto, Ontario, Canada.; ^4^ Dalla Lana School of Public Health, University of Toronto, Toronto, Ontario, Canada.

**Keywords:** generalizability, longitudinal cohort study, outcome measures, traumatic brain injury, Traumatic Brain Injury Model Systems

## Abstract

The Traumatic Brain Injury Model Systems (TBIMS) is the largest longitudinal TBI data set in the world. Our study reviews the works using TBIMS data for analysis in the last 5 years. A search (2015–2020) was conducted across PubMed, EMBASE, and Google Scholar for studies that used the National Institute on Disability, Independent Living and Rehabilitation Research NIDILRR/VA-TBIMS data. Search terms were as follows: [“TBIMS” national database] within PubMed and Google Scholar, and [“TBIMS” AND national AND database] on EMBASE. Data sources, study foci (in terms of data processing and outcomes), study outcomes, and follow-up information usage were collected to categorize the studies included in this review. Variable usage in terms of TBIMS' form-based variable groups and limitations from each study were also noted. Assessment was made on how TBIMS' objectives were met by the studies. Of the 74 articles reviewed, 23 used TBIMS along with other data sets. Fifty-four studies focused on specific outcome measures only, 6 assessed data aspects as a major focus, and 13 explored both. Sample sizes of the included studies ranged from 11 to 15,835. Forty-two of the 60 longitudinal studies assessed follow-up from 1 to 5 years, and 15 studies used 10 to 25 years of the same. Prominent variable groups as outcome measures were “Employment,” “FIM,” “DRS,” “PART-O,” “Satisfaction with Life,” “PHQ-9,” and “GOS-E.” Limited numbers of studies were published regarding tobacco consumption, the Brief Test of Adult Cognition by Telephone (BTACT), the Supervision Rating Scale (SRS), general health, and comorbidities as variables of interest. Generalizability was the most significant limitation mentioned by the studies. The TBIMS is a rich resource for large-sample longitudinal analyses of various TBI outcomes. Future efforts should focus on under-utilized variables and improving generalizability by validation of results across large-scale TBI data sets to better understand the heterogeneity of TBI.

## Introduction

Well-performed longitudinal studies in medicine can contribute greatly to understanding the clinical course, development of, and risk factors for diseases, along with long-term treatment outcomes. However, these studies are susceptible to incomplete follow-up and participant attrition over time.^1^ Longitudinal studies also possess high temporal and financial demands, and often face challenges in identifying causal exposure-outcome relations^1^. Multi-centric longitudinal studies facilitate quicker recruitment rates of more participants, which increases result representation in a population.^2^

A number of such studies have been curated, including the United Kingdom's Trauma Audit & Research Network (TARN)^3^ and the Collaborative European NeuroTrauma Effectiveness Research in Traumatic Brain Injury (CENTER-TBI).^4^ Multi-centric longitudinal studies based in the United States include: the Multicenter Osteoarthritis Study (MOST),^5^ Multicenter AIDS Cohort Study (MACS),^6^ Burn Model Systems (BMS), Spinal Cord Injury (SCI) Model Systems, Traumatic Brain Injury Model Systems National Database (TBIMS-NDB), and the Veteran Affairs (VA)-TBIMS.^7^ As longitudinal data sets encourage and facilitate the exploration of various research questions, the aforementioned data sets provide the basis for a large yield of publications. However, it can be difficult to gauge the utility, past accomplishments, current challenges, trends, clinical activity, and knowledge gaps that still exist from work on specific data sets.

The TBIMS-NDB is the largest longitudinal database for traumatic brain injury (TBI) in the world, funded by the National Institute on Disability, Independent Living and Rehabilitation Research (NIDILRR), U.S. Department of Health and Human Services since 1987.^8^ It is therefore also referred to as the NIDILRR TBIMS.^8^ The NIDILRR TBIMS was developed with the aims of providing a basis for comparison with other data sets, assessing clinical courses of patients with TBI, and informing on recovery and long-term outcomes of patients with TBI.^8^

Participants in the data set have met at least one of the following criteria set by TBIMS: a Glasgow Coma Scale (GCS) score in the emergency department below 13, post-traumatic amnesia (PTA) longer than 24 h, intracranial neuroimaging abnormalities, or a loss of consciousness (LOC) longer than 30 min.^8,9^ In addition, participants must be 16 years of age or older, and have been treated for inpatient rehabilitation at one of the 16 participating centers in the United States.^8^ The NIDILRR TBIMS database records demographic information; pre-injury history; long-term medical, social, and community reintegration; daily living; employment outcomes; the degree of disability associated with injury; and resources required.^10^ The database also includes each patient's clinical course from the time of injury to acute care/rehabilitation discharge and their long-term recovery outcomes at follow-up.^10^ Long-term follow-up data are collected at 1, 2, and 5 years, and every 5 years thereafter. For some, follow-up data of up to 30-years post-injury has been recorded. As of December 2019, information for 17,317 individuals has been entered into the NIDILRR TBIMS.^10^ Recent research has shown that the database is representative of American individuals who experience a TBI requiring hospitalization and inpatient rehabilitation.^10,11^

Beginning in 2008, the NIDILRR also partnered with the Department of Veteran Affairs through Congressional mandate, enabling the assessment of TBI recovery stages and outcomes specifically for veterans and service members admitted to one of the five polytrauma rehabilitation centers (PRCs) included in the database.^12^ Unlike the NIDILRR TBIMS, the VA-TBIMS database collects information on all veterans and service members from around the world who have experienced a TBI of any severity, from mild to severe.^12^ This is in contrast to the NIDILRR TBIMS database, which limits participant inclusion to moderate-severe TBIs and specified cases of mild TBI, based in the United States.^8,12^ Similarities across the two databases are attributable to modeling the VA-TBIMS after the NIDILRR TBIMS.^12^ Modifications were made to address cohort-specific variables related to combat, deployment, injury severity, years of service, the assessment of post-traumatic stress disorder (PTSD) symptoms, and patient care upon admission to rehabilitation.^12^

Although recent reviews of TBI-related neurological outcomes,^13^ suicide attempt and ideation,^14^ and sleep dysfunction comorbidities^15^ have included studies that used NIDILRR/VA-TBIMS data, our focus was to specifically assess how the NIDILRR/VA-TBIMS data have been used in recent TBI-related studies (2015–2020). We conducted a narrative review, highlighting the diversity of studies related to the data set and areas, outcomes, and methodologies of focus in the research communities regarding TBI. We also summarized the limitations noted by each study, and assessed the NIDILRR and VA-TBIMS databases' abilities to achieve their stated objectives. Finally, we identified areas of future research for the TBIMS databases and other multi-centric, longitudinal data sets.

## Methods

**FIG. 1.**
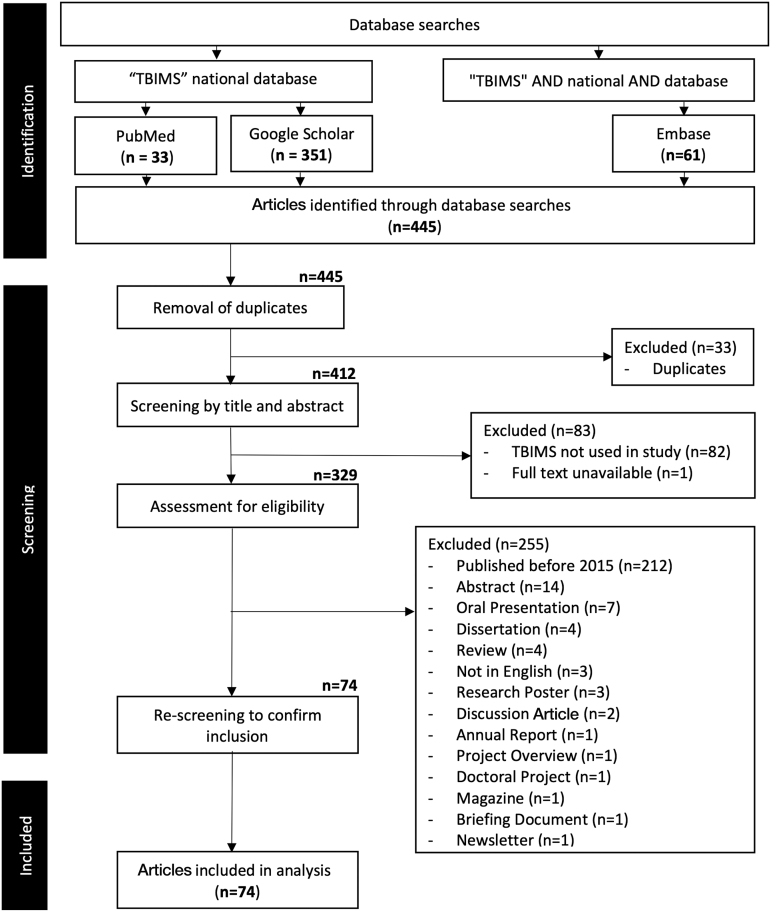
Search strategy. Flowchart describing methods for inclusion of studies, with detailed exclusions.

PubMed, EMBASE, and Google Scholar were searched on June 3, 2020 for studies that used the NIDILRR/VA-TBIMS. Initially, no time-frame limitations or search filters were used, to allow for a thorough scope of TBIMS utility. Search terms were as follows: [“TBIMS” national database] within PubMed and Google Scholar, and [“TBIMS” AND national AND database] on EMBASE. Although over 100 studies have been conducted from the beginning of TBIMS data collection, we elected to focus on recent publications (2015–2020). Full details for inclusion/exclusions are shown in Figure 1. As this article is a review of previously published works related to TBIMS and did not use human subject information from the TBIMS data set itself, ethics board approval was not required.

Information was collected regarding the first author, publication year, sources of data analysed (NIDILRR/VA-TBIMS or additional sources), cohort specifics, study objectives, measurements or variables used, and the use of follow-up information. Three principal aspects of the studies were quantified, as follows:

### Categorization based on the usage, focus, and applications of TBIMS data

We categorized studies in four different ways, guiding our comparison through use of an overarching question for each mode of comparison:

#### Data source (*Was NIDILRR/VA-TBIMS the only database used in the study?*)

We determined if a study had utilized only NIDILRR/VA-TBIMS variables, or collected additional information outside of the TBIMS databases. We also noted among the studies that utilized NIDILRR/VA-TBIMS if a specific subset of data was used based on the research question.

#### Study focus and applications (*What was the focus and application of the study?*)

We noted the usage and applications of the data in published works. Specifically, we noted if a study focused on:

(a) Data related aspects such as data quality, data mining, alternate or improved ways of data processing/analytical methodology as a contribution of the study.
(b) Evaluation of specific measurements of interest.
(c) Both (a) and (b).

#### Study outcomes (*What measurements were assessed/reported as an outcome of interest for the studies?*)

We noted the broad measurements that were used as outcomes of interest in the studies. In addition, we noted sample sizes in relation to each study's outcome(s) of interest.

#### Usage of follow-up information (*Was follow-up data used in the study?*)

To determine this, we checked the intervals of follow-up data that were used in each study.

### Variable groups of interest

Upon their mentioning, the use of specific variable groups from the NIDILRR TBIMS was recorded for each study. Variable groups were obtained from the NIDILRR TBIMS Syllabus, as of August 2020. There were two possible sources from which variables in the variable groups were obtained: Form 1 and Form 2.^16^ Form 1 included information collected at the time of injury, acute care, and rehabilitation.^16^ Form 2 included information that was obtained at follow-up intervals of 1, 2, and 5 years, and every 5 years following.^16^ To reduce the number of variable groups for the ease of analysis, we merged related variable groups to form “composite variable groups.” Frequently used VA-TBIMS-specific variables and non-TBIMS variables that were used in studies were also noted.

In keeping record of variable group usage for each study, we also explored how these variable groups were used: either being simply included in the study, or being used as a main outcome measure. We defined an outcome measure as a dependent variable, resulting from another factor. Additionally, outcome measures were noted when explicitly listed as such in a given study. Tracking variable group usage allowed the assessment of their patterns and total frequencies across studies.

### Limitations

The limitations of each study were noted and compared to identify trends. Limitations, which were a result of the study design, were differentiated from those brought on to a study in using TBIMS data.

Finally, an additional assessment was made, based on the studies reviewed, to determine if the objectives of the NIDILRR/VA-TBIMS data sets had been fulfilled at present.

## Results

Of the 74 studies included in the review, *n* = 15, 11, 13, 17, 14, and 4 articles were published respectively, in the consecutive years from 2015 through 2020.

### Categorization based on the usage, focus, and applications of the data

**FIG. 2.**
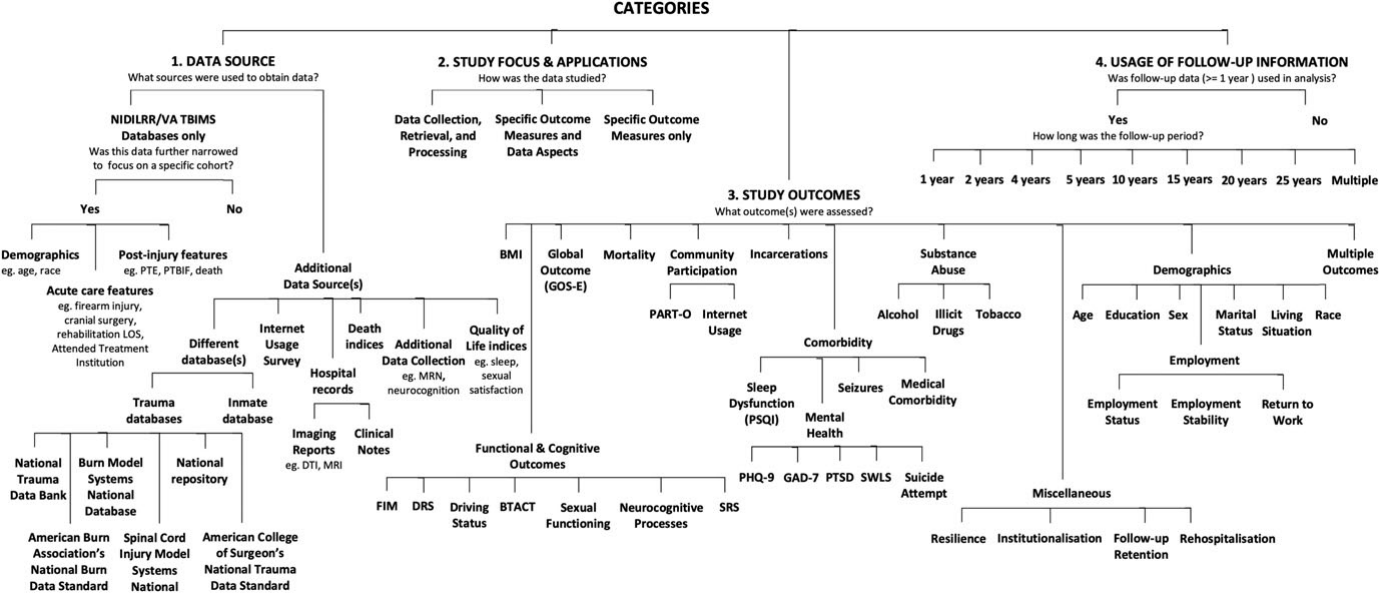
Categories. The four means of categorizing studies are shown. BMI, body mass index; BTACT, Brief Test of Adult Cognition by Telephone; DRS, Disability Rating Scale; DTI, diffusion tensor imaging; FIM, Functional Independence Measure; GAD-7, Generalized Anxiety Disorder-7; GOS-E, Glasgow Outcome Scale-Extended; LOS, length of stay; MRI, magnetic resonance imaging; MRN, medical record number; PART-O, Participation Assessment with Recombined Tools-Objective; PHQ-9, Patient Health Questionnaire-9; PSQI, Pittsburgh Sleep Quality Index; PTBIF, post-traumatic brain injury fatigue; PTE, post-traumatic epilepsy; PTSD, post-traumatic stress disorder; SRS, Supervision Rating Scale; SWLS, Satisfaction with Life Scale.

The outline for categorization is shown in Figure 2.

#### Data source

Altogether, 23 studies gathered information from additional sources. Additional information from other databases included relevant information from surveys,^17–24^ hospital records,^25–29^ death indices,^30–33^ quality of life indices,^32,34,35^ measures of neurocognition,^36,37^ and national geographic information systems.^38^ Moreover, external validation cohorts were collected.^30,39^ The remaining 51 articles exclusively used NIDILRR/VA-TBIMS data. Twelve of these 51 studies focused further on a specific cohort within the data set. Examples of these specific cohorts include mortality,^40^ participants of a specified age range,^23,36,41–44^ and patients who underwent cranial procedures,^45^ developed post-injury conditions such as post-traumatic brain injury fatigue (PTBIF),^34^ post-traumatic confusional state (PTCS),^37^ or disorders of consciousness (DOC).^28^ It is important to note that each study's objective(s) determined how the TBIMS data were used, and whether other data sources were required to conduct their analysis.

#### Study focus and applications

Three sub-categories were defined as follows:

##### Data collection, retrieval, and processing

Six studies focused on data-related aspects. Examples include: a descriptive summary of the various characteristics and outcomes in the VA-TBIMS,^12^ an assessment of the challenges present in standardization of trauma data,^19^ and a comparison of VA and NIDILRR TBIMS data sets.^12^ Two studies developed techniques to link NIDILRR TBIMS data with that of a trauma registry using probabilistic matching.^17,39^ Another study validated the utility of iterative proportional fitting to weigh TBIMS and align population estimates and parameters.^46^

##### Specific outcome measures only

Fifty-four articles focused on characterizing specific outcomes either by descriptive analysis, exploratory analysis (including prognostication), or longitudinal analysis using statistical techniques. The details of these outcome measures are discussed below in the “Study outcomes” category.

##### Specific outcome measures with significant data aspects

Thirteen articles focused on specific outcome measures and had additional motivation for focusing on alternative or improved methodologies for data analysis These studies include the application of multi-variate Rasch analysis for Functional Independence Measure (FIM) subscales, as the subscales are correlated,^47^ modifications on item-scoring in the Participation Assessment with Recombined Tools-Objective (PART-O) based on concerns in literature,^48^ and the usage of propensity matching replacement technique for balancing baseline characteristics in patients having craniectomy and craniotomy.^45^ In addition, usage of cross-lagged structural equation modeling was assessed for multiple temporally dependent and bidirectional relationships in the context of substance abuse and employment,^21^ and FIM and mental health correlations.^49^ Test-retest reliability analysis of outcome measures was performed by Bogner and colleagues.^50^ Both regression and categorization of rehospitalization using generalized linear modeling was performed by Erler and associates.^51^ Utilization of hierarchical linear modeling (HLM) for the trajectory of life satisfaction was investigated by Williamson and co-workers.^52^ Internally validated prediction models based on logistic regression were developed for prognostication of institutionalization^53^ and seizures.^54^ External validation of survival models was performed by Brooks and colleagues.^30^ Other data-driven approaches (machine learning) include decision tree-based prediction of long-term global outcomes^55^ and employment.^44^

#### Study outcomes

This category is related to the sub-category above, “Specific outcome measures only.” Functional, cognitive, and global outcomes, body mass index (BMI), mortality, community participation, incarcerations, health conditions, substance use, demographics (age, sex, education, employment, race, marital status, and living situation), and other variable groups (institutionalization, rehospitalization, resilience, and follow-up retention) were included/reported as outcome measures. There were areas of overlap within this category itself, as several studies had multiple outcome measures of focus. The overall diversity in applications of the TBIMS data sets was emphasized upon this further categorization of studies, as an extension of the “Study focus and applications” category above.

#### Usage of follow-up information

Sixty studies (81%) had a longitudinal design, whereas 14 did not use longitudinal data. Three studies^27,33,37^ had a follow-up period of less than 1 year. One study used up to 25 years of follow-up data.^56^ Seven studies included participants with up to 20 years of follow-up. Another study included participants with up to 15 years of follow-up.^48^ Six studies included participants with up to 10 years of follow-up data. However, within the longitudinal studies, 42 studies (70%) were conducted between 1 and 5 years. Among these 42 studies, 19 studies (45%) had a 5-year follow-up period, 11 studies (26%) used 2-year follow-up information, and 12 studies (29%) used data with 1-year of follow-up.

### Variable groups of interest

The TBIMS Syllabus accounts for 148 variable groups: 71 are from Form 1, and 77 are from Form 2.^16^ In many cases, variable groups are comprised of multiple variables within each group.^16^ Variable groups that yielded similar information were merged to form composite variable groups. In total, 54 variable groups were used in our analysis, with 28 newly constructed composite variable groups and 26 unchanged variable groups from the TBIMS Syllabus. The 54 variable groups were separated into three types: Sociodemographic (collected from Form 1 and/or Form 2) comprising 15 variable groups, Other Variable Groups (collected from Form 1 and/or Form 2) consisting of 25 variable groups, and Variable Groups Unique to Form 2, containing the remaining 14 variable groups. Details for both variable group-typing and composite variable constructs are shown in Supplementary Table S1, under the tab, “Variable Composition.”

**FIG. 3.**
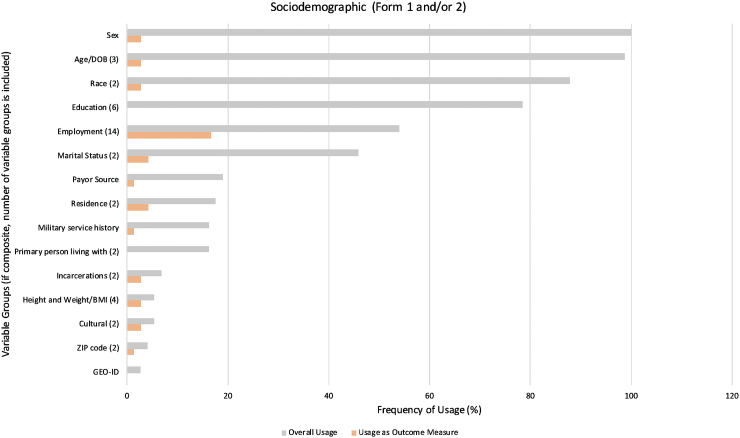
Usage of sociodemographic variable groups (Forms 1 and/or 2). Frequency of usage for each variable group overall (gray) and as an outcome measure (orange) were compared across sociodemographic variable groups found in Form 1 and/or 2. The number of variables groups that were combined to form composite variable groups is provided in parentheses next to each composite variable group. BMI; body mass index; DOB, date of birth; GEO-ID, geographic identifier; ZIP, zone improvement plan.

**FIG. 4.**
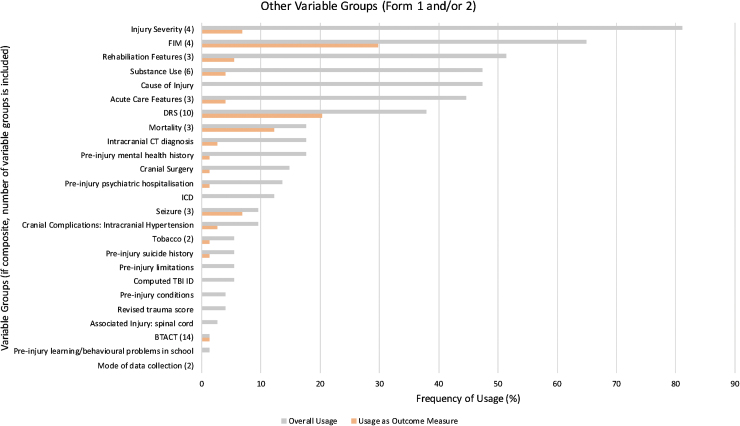
Usage of other variable groups (Forms 1 and/or 2). Frequency of usage for each variable group overall (gray) and as an outcome measure (orange) were compared across non-sociodemographic variable groups found in Form 1 and/or 2. The number of variables groups that were combined to form composite variable groups is provided in parentheses next to each composite variable group. ICD, International Classification of Diseases; FIM, Functional Independence Measure; CT, computed tomography; DRS, Disability Rating Scale; BTACT, Brief Test of Adult Cognition by Telephone; TBI ID, traumatic brain injury identity document.

**FIG. 5.**
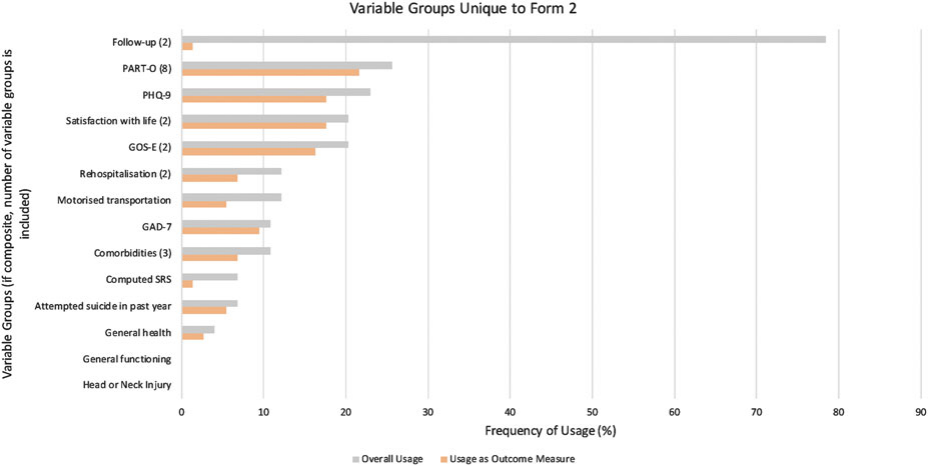
Usage of variable groups unique to Form 2. Frequency of usage for each variable group overall (gray) and as an outcome measure (orange) were compared across non-sociodemographic variable groups found within Form 2. The number of variables groups that were combined to form composite variable groups is provided in parentheses next to each composite variable groups. GAD-7, Generalized Anxiety Disorder-7; GOS-E, Glasgow Outcome Scale-Extended; PART-O, Participation Assessment with Recombined Tools-Objective; PHQ-9, Patient Health Questionnaire-9; SRS, Supervision Rating Scale.

The names and frequencies of use for each variable group are shown in Figures 3, 4, and 5, displaying variable group usage, both overall and as an outcome measure, within their respective types. Worthy of note in Figure 3: several variable groups were frequently collected yet not used commonly as an outcome measure, such as “Sex,” “Age,” and “Race.” Non-sociodemographic variable groups with the highest overall frequency of usage included “Injury Severity” (81%) in Figure 4, and “Follow up” (78%) in Figure 5.

With regards to usage as an outcome measure, “Employment” was most frequently used within the Sociodemographic variable groups type, being in 12 studies (17%; as shown in Fig. 3). According to Figure 4, the greatest frequencies of usage as outcome measures were the “FIM” (30%) and “DRS” (20%). Outcome measures in Figure 5 that occurred most commonly included “PART-O” (22%), “Satisfaction with Life” (18%), “PHQ-9” (Patient Health Questionnaire-9; 18%), and “GOS-E” (Glasgow Outcome Scale-Extended; 16%).

As noted previously, 23 studies used additional data sources to obtain information not provided by the NIDILRR/VA-TBIMS data sets. Of these, 13 studies used the external information as a primary outcome measure in conducting their research. Information was collected from surveys for cognitive assessments,^36,37^ Internet usage,^24^ resilience,^33,35^ sleep quality,^34^ and sexual satisfaction.^32^ Other trauma registries,^18–21,23,30,38,39,57^ hospital records,^25–29^ death indices,^22,30,31^ and inmate databases^22^ were used to collect additional information. In some cases, studies used VA-TBIMS-specific variables including: PTSD variables^42,58^ and the PTSD checklist-civilian (PCL-C),^59–63^ deployment status at the time of injury,^42,60,64^ the Neurobehavioral Symptom Inventory (NSI),^61^ the Mayo-Portland Adaptability Inventory-4 (MPAI-4),^63^ and blast injury.^64^

In summary, certain variables found more usage than the others. NIDILRR/VA TBIMS data sets were used in conjunction with other types of variables outside the TBIMS forms.

### Limitations noted in studies

**FIG. 6.**
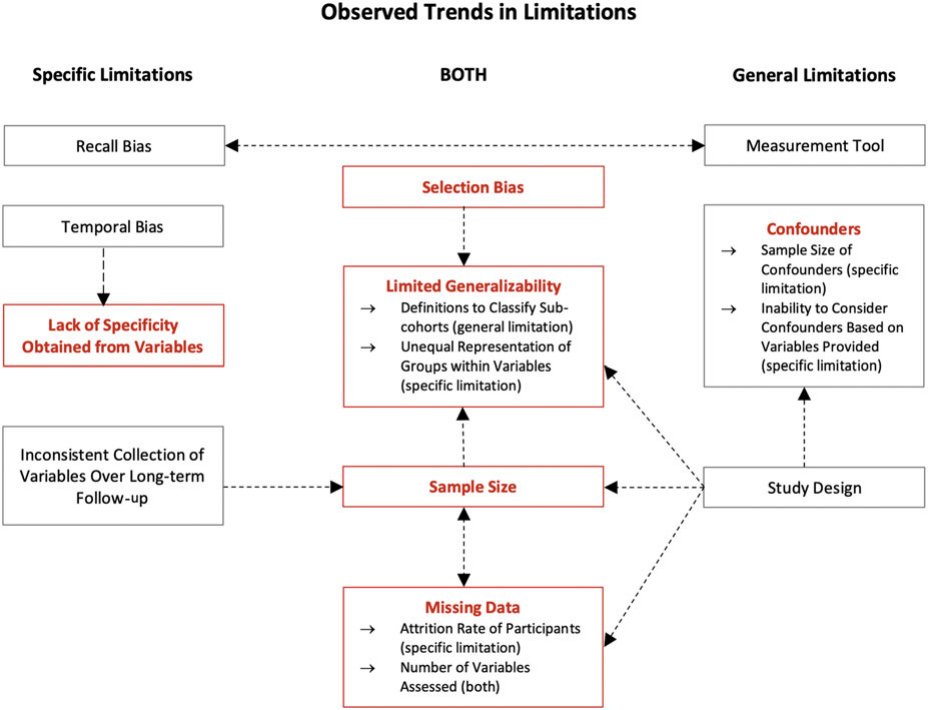
Limitation trends. Arrows display correlations between various limitations. Direction of causality is indicated by arrowheads, being single or reciprocal. Limitations in red are the main limitations discussed with more details in this article. Limitations in gray represent other limitations.

Study limitations were attributable to either the design of the study or the use of NIDILRR/VA-TBIMS data for analyses. Limitations related to generalizability, biases, missing data, sample sizes, variables provided by the data set, and confounders were seen across most studies. Often, these limitations are interrelated. The interrelations are outlined in Figure 6.

#### Selection bias and limited generalizability

Forty-six articles (62%) reported selection bias in their “Limitations” section. As the NIDILRR enrollment criteria for both TBIMS data sets possess restrictions relating to age and TBI severity, all studies that used TBIMS data are generalizable primarily to NIDILRR/VA-TBIMS study participants.^8^ Although through recent research^8,10,11^ the database has proven representative of individuals who have experienced a TBI severe enough to require hospitalization and inpatient rehabilitation in the United States, the results of studies that used the NIDILRR TBIMS are generalizable to a specific patient population that has endured a moderate-severe TBI as per their criteria.

Results from studies that used the VA-TBIMS data will be specifically applicable to the veteran population. Studies that used information from only one of the 16 hospitals in their analysis lacked generalizability due to their use of single-center data.^17,39,65^ In addition, difficulties to generalizability were seen in studies with specific foci, having limited sample sizes available that satisfied their inclusion criteria.^14,18,20,23,27,28,32–34,38,40,50,53,55,57–62,64,66–71^ Thirty of 74 articles (41%) reported their limited ability to generalize to the overall TBI population, including those under-represented in the TBIMS data sets due to loss of follow-up or by database collection methods. Other factors impacting generalizability include sample size and missing data, which is a limitation in itself.^14,18,20,23,27,28,32–34,38,40,50,53,55,57–62,64,66–71^ In addition, certain variable groups such as “race” were under- or over-representative of the U.S. population overall, therefore serving as a limitation to generalizability in results due to the unequal representation of groups within variables.^60^ In many studies, cohorts were dichotomized into sub-cohorts based on specific thresholds^21,23,28–31,34,37,41,45,55,60,61,68,69,72,73^; therefore caution is required when applying these findings to a more general population.

#### Missing data and sample size

As the TBIMS databases collect follow-up information at 1, 2, and 5 years, and every 5 years thereafter, it is common to have incomplete data on patients due to participant withdrawal or loss of follow-up. In addition, missing information that occurred outside of the time period that a given variable had been collected/included by the data set consequentially limited some studies to a certain time period (e.g., height and weight variable collection began in 2013).^59^ Some studies noted that the attrition rate of participants could affect the quality of data collected as a result of the long-term follow-up nature of the TBIMS data sets,^51,74^ whereas others were limited by the number of variables from the TBIMS data sets that could be assessed due to missing data over long-term follow-up periods.^23,70,74,75^

#### Lack of variable specificity

Despite the wide coverage of variables, several studies that have used TBIMS data indicated that the amount of detail provided by the variables collected could potentially be problematic when interpreting results. Examples of variables include collection of general information with a lack of details^70^ and dichotomized data (yes/no)^41^ provided in the data set.

#### Confounders

Confounders varied depending on each study focus, so it is important to note possible confounding variables upon assessment of a study's results. For example, a participant's geographical location and their race must be considered as possible confounders when comparing FIM communication ratings for English versus non-English speakers.^72^ Similarly, when assessing long-term functional outcomes in relation to DOC due to TBI, any change to health policies over time could be an important confounder to consider.^28^ Several studies found it impossible to account for all confounders in a given study due to an absence of necessary variables collected in the database, or lack of adequate sample sizes present to consider a variable.^25,76^

#### Other limitations

Upon assessing the limitations noted within the articles, additional trends in limitations across the 74 studies were identified.

##### Study design

The methodology (analytic models used, outcomes assessed, selected variables for analysis, or study nature itself (retrospective, prospective, cross-sectional, longitudinal, etc.) was often mentioned as a limitation in studies. The impact of the study design was seen to influence confounders, and result in missing data.

##### Temporal bias

Temporal bias was relevant to studies that lacked information regarding timing of an outcome (e.g., ischaemic stroke^25^).

##### Measurement tools used

Various measurement tools were mentioned as limitations in studies, being compared to other modes of assessment to define outcomes. Examples of measures mentioned as limitations include: the GCS to measure initial head injury severity,^17^ FIM as a functional and cognitive measure,^34,71,72^ diffusion tensor imaging (DTI) scanner resolution,^27^ GOS-E, and DRS.^77,78^ As there are several different modes of measuring variables across institutions, this leads to slight differences in the information being collected.

##### Recall bias

Recall bias was seen across studies due to the nature of many variables in the TBIMS data set. Particularly, some follow-up information from variable groups in Form 2 (e.g., seizures, substance abuse) was susceptible to poor recall and poor self-awareness, which could impact result reliability.

##### Inconsistent collection of variables over long-term follow-up

As mentioned previously, certain variables were limited with regards to the timeframe that data were collected, impacting a study's ability to conduct a long-term follow-up on specific outcomes,^59^ or limiting the years in which specific variables or outcomes could be assessed in relation to one another.^14^

All limitations are important to consider when interpreting the results of a specific study. A general review and discussion of data and analysis related can be found in recent works.^79,80^

##### Meeting set objectives

Three objectives were stated by the NIDILRR TBIMS:

1. “establish a basis for comparison with other data sources,”
2. “assess the clinical course of individuals with TBI from time of injury through discharge from acute care and rehabilitation,” and
3. “evaluate recovery and long-term outcomes of patients with TBI.”^8^

In addressing these goals, we have found that:

1. The aim to establish a basis for data comparison with other data sets appears well under way, with recent studies having conducted comparisons and linkages to confirm compatibility with other trauma registries. In the above section “Study focus and applications” , comparisons of data aspects were seen in a select number of studies through using additional data.^12,17,19,39^
2. Assessment of the clinical course for individuals with TBI, between the time of injury and discharge from acute care and rehabilitation, was not seen to be performed by any of the studies that we reviewed from the last 5 years.
3. Several studies focused on either recovery aspects or long-term outcomes of patients with TBI, whether it be regarding long-term employment^21,23,36,38,42–44,57,60,68^ or functional outcome measures.^27,28,45,49,52,66,81,82^ As per the subsections “Study focus and applications” and “Study outcomes” of the “Results” section, there was more emphasis on usage of the follow-up data from 1 to 5 years.

## Areas Requiring Further Exploration Using TBIMS

Sociodemographic and injury variables have been used to account for confounders or serve as possible predictors in several studies. These variables could be explored further in studies focusing on outcomes such as substance use or to assess attrition and missing data.

Other variable groups that have not received much attention but are collected in this data set include, but are not limited to: tobacco consumption, the Brief Test of Adult Cognition by Telephone (BTACT), the Supervision Rating Scale (SRS), general health, and comorbidities. Exploring these additional aspects of TBIMS can serve to optimize usage of TBI data that currently exist, while also progressing with TBI research.

### Large data analytics

The sample size of each study ranged from 11^27^ to 15,835.^40^ The following study variables have the largest sample sizes and could be exploited to a great degree: acute ischemic stroke (*n =* 6,488 participants),^25^ suicidality (*n =* 3,575),^18^ employment (smallest sample size: *n =* 2,784, largest sample size: *n =* 7,867),^21,36,44,68^ driving and participation (*n =* 2,456),^83^ global disability and supervision (*n =* 4624),^81^ Glasgow Outcome Scale and hospitalization frequency for seizures (*n =* 6,111),^29^ mortality (*n =* 7,315),^30^ BMI (*n =* 7,827),^84^ long-term global outcomes (*n =* 10,125),^55^ institutionalization (*n =* 7,219),^53^ and return to productivity (*n =* 2,542).^57^ The large-scale nature of TBIMS lends itself to large data analytical techniques such as machine learning. However, usage of data-driven approaches/machine learning on TBIMS data has been low, considering the rapid machine learning or artificial intelligence-based advancement in medicine.^85^ Thus, potential studies using TBIMS data can benefit from machine learning-based techniques.

## Discussion

TBIMS is the largest multi-site (16 centers) longitudinal TBI data set in the world and is funded by the NIDILRR, U.S. Department of Health and Human Services since initiation in 1987. In 2008, the VA-TBIMS was formed by VA-specific longitudinal multi-centers (5 centers), contributing to the understanding of TBI in veterans. Two other multi-site longitudinal data sets funded by the NIDILRR program include the SCI Model Systems (SCIMS; beginning in 1970, with data collection currently from 14 centers) and the Burn Model Systems (BMS; beginning in 1994, currently collecting from 4 centers). In Europe, TARN has collected longitudinal trauma data since 1990 and currently involves 220 contributing centers. As longitudinal data sets continue to grow in size and studies conducted, reviews are conducted on those for a greater understanding of the study trends, clinical activity, and accomplishments. Several of such reviews were present for SCIMS, BMS, and TARN.^3,86–90^ However, reviews on the diverse applications and frequently used information from the TBIMS have not been conducted. We have addressed this in our study.

### Categorization of TBIMS studies based on the usage, focus, and applications of the data

#### Data source

Fifty-one of the 74 studies (69%) used the TBIMS data on its own to assess a specific outcome. Although only using one data source, together these studies covered a wide range of outcomes. With such variety in studies conducted, a better overall understanding of TBI patient characteristics, treatments, and long-term outcomes has been achieved. The 23 remaining studies (31%) required additional information from other sources to attain their objectives. However, acknowledging that the cross-linking of registrars was possible among various databases with TBIMS data sets suggests that the TBIMS data have a high level of compatibility with other networks. This is similar to cross-linkages conducted using other longitudinal data sets such as TARN^91^ and CENTER-TBI.^92^

#### Study focus and applications

Applications of the TBIMS data were very versatile, as it could be used in both clinical and data-related aspect analyses. Six studies (8%) focused on data-related aspects, 54 studies (73%) focused on clinical or social outcome measures, and 13 studies (18%) focused on both the TBIMS data aspects and outcome measures.

#### Usage of follow-up information

Only 15 studies (20%) were conducted with greater than 10 years of follow-up information and one study used 25 years of follow-up data. In comparison, several works used the complete follow-up range in the MOST data set.^93–95^ As TBIMS data are still continually being collected, there is hope that more studies will use 10 or more years of follow-up data in the future. Similar outlooks are seen with other longitudinal data sets, including the SCIMS and BMS, which are also currently collecting data potentiating more long-term follow-up studies to be undertaken in the future.^96,97^

### Variable groups of interest

The most commonly used variables overall were “Sex,” “Age,” “Race,” “Injury Severity,” and “Follow Up.” These variables were often used to account for confounders or derive cohorts in the results rather than for direct assessments in correlations to outcome measures. Variable groups collected most frequently as outcome measures included “Employment,” “FIM,” “DRS,” “PART-O,” “Satisfaction with Life,” “PHQ-9,” and “GOS-E.” By identifying the variables that have been used most frequently in the analyses of recent studies, knowledge of such patterns could serve as a useful guide for selecting components to include during the development of future TBI data sets.

### Limitation trends

Limitations to generalizability were most abundantly recognized across studies. However, some articles were able to conduct external validation using other data sets.^30,39^ Future studies should aim to conduct validation using publicly accessible data sets, and not be restricted exclusively to TBIMS data in their analysis. There are several available data sets, for instance TARN, for which external validation has often been performed.^98,99^

### Meeting data set objectives and further exploration

The TBIMS data sets have had great success in achieving their study objectives, as several recent studies have compared NIDILRR/VA-TBIMS data with other data sets. In addition, assessments of long-term outcomes and recovery for patients with TBI have been made possible by using TBIMS data. Although the database also aimed to serve as a tool to examine the clinical course of individuals who sustain a TBI, there have not been recent studies to address that for the time between injury and discharge from acute care.

Long-term assessments in TBI research have had great focus on employment, functional outcomes, and quality-of-life measures. However, the variety of data found within the TBIMS data set provides opportunities to expand the knowledge base using these data. Following the identification of variable groups that were most often used across the studies in the subsection “Variable Groups of Interest” of the “Results” section, research gaps related to variable groups that have not been conventionally seen under the spotlight in TBI research were identified. Examples of potential future works could focus on tobacco consumption, the extent of caregiver assistance received via the SRS, or comorbidities, which were all mentioned previously in the section, “Areas Requiring Further Exploration Using TBIMS.”

There are also promising future opportunities to use machine learning and data-driven approaches to fully explore the rich data found in the TBIMS in ways that have yet to be used.

## Conclusion

We conducted a review of research articles that used NIDILRR TBIMS/VA-TBIMS data. Seventy-four major articles based on the TBIMS have been published in the last 5 years, representing a significant contribution of this important data set. Studies have capitalized on TBIMS longitudinal nature and outcomes such as the GOS, FIM, and employment but relatively paid limited attention certain groups of variables such as tobacco consumption, SRS, and comorbidities. Despite the combination with other data sets, there remain concerns about limited generalizability of results due to missing data and attrition. There remains the great possibility to use data-driven approaches to identify new trends, examine generalizability, and explore longer-term prognostic studies.

## Supplementary Material

Supplemental data

## Abbreviations Used

BMI: body mass index
BMS: Burn Model Systems
BTACT: Brief Test of Adult Cognition by Telephone
CENTER-TBI: Collaborative European NeuroTrauma Effectiveness Research in Traumatic Brain Injury
CT: computed tomography
DOB: date of birth
DOC: disorders of consciousness
DRS: Disability Rating Scale
DTI: diffusion tensor imaging
FIM: Functional Independence Measure
GCS: Glasgow Coma Scale
GEO-ID: geographic identifier
GOS-E: Glasgow Outcome Scale-Extended
HLM: hierarchical linear modeling
ICD: International Classification of Diseases
LOC: loss of consciousness
MACS: Multicenter AIDS Cohort Study
MOST: Multicenter Osteoarthritis Study
MPAI-4: Mayo-Portland Adaptability Inventory-4
MRN: medical record number
NIDILRR: National Institute on Disability, Independent Living and Rehabilitation Research
NSI: Neurobehavioral Symptom Inventory
PART-O: Participation Assessment with Recombined Tools-Objective
PCL-C: PTSD checklist-civilian
PCR: polytrauma rehabilitation center
PHQ-9: Patient Health Questionnaire-9
PSQI: Pittsburgh Sleep Quality Index
PTA: post-traumatic amnesia
PTBIF: post-traumatic brain injury fatigue
PTCS: post-traumatic confusional state
PTE: post-traumatic epilepsy
PTSD: post-traumatic stress disorder
SCI: spinal cord injury
SCIMS: SCI Model Systems
SRS: Supervision Rating Scale
TARN: Trauma Audit & Research Network
TBI: traumatic brain injury
TBI ID: traumatic brain injury identity document
TBIMS: Traumatic Brain Injury Model Systems
TBIMS-NDB: Traumatic Brain Injury Model Systems National Database
VA: Veterans Affairs
VA-TBIMS: Veterans Affairs Traumatic Brain Injury Model Systems
ZIP: zone improvement plan
